# Tuberculosis remains a major burden in systemic lupus erythematosus patients in Durban, South Africa

**DOI:** 10.3389/fmed.2023.1118390

**Published:** 2023-03-01

**Authors:** Khaled Mohamed Sefow Al-arbi, Nombulelo P. Magula, Girish M. Mody

**Affiliations:** ^1^Department of Rheumatology, Nelson R Mandela School of Medicine, University of KwaZulu-Natal and Inkosi Albert Luthuli Central Hospital, Durban, South Africa; ^2^Division of Internal Medicine, Nelson R Mandela School of Medicine, University of KwaZulu-Natal, Durban, South Africa

**Keywords:** tuberculosis, systemic lupus erythematosus, mortality, infection, corticosteroids, Black African people, Indian people

## Abstract

**Objective:**

Infections are common in systemic lupus erythematosus (SLE), with tuberculosis (TB) being important in an endemic environment. We studied the prevalence and spectrum of TB in SLE in Durban, South Africa.

**Methods:**

A medical records review of SLE patients seen over 13-year period, and the demographic data, clinical manifestations, laboratory findings, treatment and outcome were noted.

**Results:**

There were 512 SLE patients and 72 (14.1%) had TB. Thirty (41.7%) had pulmonary TB (PTB) and 42 (58.3%) had extra-pulmonary TB (EPTB). The prevalence of TB among the different ethnic groups was 36/282 (12.8%) for Indian people, 29/184 (15.8%) Black African people, 7/26 (26.9%) admixed African people and none among the 18 White people. Comparison of the 72 SLE-TB patients with 72 SLE controls showed no difference in gender, age at SLE diagnosis and disease duration. The SLE-TB patients had a significant increase in the clinical and laboratory features of disease activity (arthritis, mucocutaneous lesions, renal involvement, vasculitis, low complement, raised ds-DNA antibodies), and cumulative prednisone use over the preceding 3 months.

Compared to PTB, the EPTB patients were significantly younger, developed TB earlier after SLE diagnosis, and had higher disease activity. The EPTB patients also had increase in features of disease activity (renal, thrombocytopenia, ds–DNA antibodies), and increase in ever use of intravenous methylprednisolone (IV-MP) and mycophenolate mofetil (MMF). On multivariate analysis, the independent risk factors for EPTB were ever use of MMF (*p* = 0.003) and IV-MP (*p* = 0.027). Analysis of the cumulative SLE criteria showed renal involvement was an independent risk factor for EPTB. The outcome was similar in both groups.

**Conclusion:**

We show an increased prevalence of TB (14.1%) and EPTB (58.3%) in SLE in an endemic area and confirm that features of disease activity and use of immunosuppressive therapy are the major risk factors. Renal involvement (as a cumulative criterion) is an independent risk factor for EPTB.

## Introduction

Although there is marked improvement in the outcome and survival in SLE, infections remain an important cause of morbidity and mortality (1). They may occur in the early or late stages of SLE, and account for about 14–50% of the hospitalizations (2, 3). In the Euro-lupus cohort of 1,000 patients, followed up for 10 years, infections accounted for 25% of the mortality (4). The increased risk of infections is attributed to disease related factors which lead to dysregulation of the immune system, and the use of immunosuppressive drugs (5).

A recent systematic review and meta-analysis on the global incidence and prevalence of tuberculosis (TB) in SLE noted an increased prevalence in Africa and countries with a high TB burden, and a lower prevalence in Europe and North America. (6). Based on a review of 46,327 patients in 35 studies, Wu et al. (6) reported an incidence of TB in SLE of 1.16 [95% confidence interval (CI) 0.69–1.93] per 100 person years and the prevalence was 3.59% (95% CI 2.57–5.02%). In clinical studies, there was a wide variation in the prevalence of TB in SLE with a lower prevalence of 0.66% in Taiwan and 1.3% in Mexico, and a higher prevalence of 17.1% in South Africa and 25% in Colombia (7–10). A review of studies with more than 50 patients, showed increased extrapulmonary TB (EPTB) in Colombia (54%), Hong Kong (67%) and Philippines (67%) (10–12).

Risk factors for the development of TB include the use of intravenous and oral corticosteroids (mean daily doses prior to the diagnosis of TB, the cumulative dose and duration of treatment), use of immunosuppressive therapy, disease activity and manifestations such as nephritis and vasculitis (8–15).

The 2022 WHO Global report on TB estimated 10.6 million new TB cases in 2021, being most common in South- East Asia (45%), Africa (23%), Western Pacific (18%), and Eastern Mediterranean (8.3%) (16). There were a smaller proportion in the Americas (2.9%) and Europe (2.2%) (16). The estimated number of global TB deaths is about 1.6 million, of whom about 187,000 were in HIV positive patients (16). South Africa has a very high burden of TB with an estimated incidence of 513 per 100,000 population (17). The high burden increases the risk of TB in patients who are immunocompromised or have autoimmune diseases and require intensive immunosuppressive therapy. Thus, this study was undertaken to determine the prevalence, clinical spectrum, risk factors and outcome of TB in SLE patients in a multi-ethnic population in a single academic center in a high TB burden environment in Durban, South Africa. We also studied the risk factors for TB in SLE patients compared to controls, risk factors for PTB vs. EPTB, and compared our findings with observations in other parts of the world.

## Methods

We reviewed the medical records of all the patients with SLE seen in the Department of Rheumatology at, Inkosi Albert Luthuli Central Hospital (IALCH) in Durban, South Africa from June 2003 to March 2016. Patients who fulfilled the Systemic Lupus International Collaborating Clinics (SLICC) criteria for SLE were selected for inclusion in the study (18). Patients with incomplete SLE, concomitant HIV infection, mixed connective tissue disease or overlapping features of another connective tissue disease such as scleroderma or inflammatory myositis, were excluded from the study.

Patients were classified as having TB if: (a) acid fast bacilli were identified on microscopy of sputum or other tissue/specimen; (b) a positive GeneXpert test result was obtained on the sputum or other specimen; (c) *Mycobacterium tuberculosis* was cultured from the sputum or other appropriate specimen; (d) the presence of histological evidence of caseating granuloma or (e) clinical diagnosis: patients in whom a diagnosis of TB was made on the basis of a combination of symptoms, clinical findings, imaging studies or the results of laboratory investigations and were treated for TB by the attending physician.

The records of the patients with SLE and TB were analyzed further. The ethnicity/racial background was defined according to the guidance by Flanagin et al. (19). The demographic data recorded included gender, age at diagnosis of SLE and the interval from SLE diagnosis to TB diagnosis. The clinical manifestations, results of laboratory tests and imaging studies, and the SLEDAI – 2 K (Systemic Lupus Erythematosus Disease activity index -2 K) at TB diagnosis were also recorded (20). The treatment recorded included whether patients received chloroquine, corticosteroids, or immunosuppressive therapy such as cyclophosphamide, methotrexate, mycophenolate mofetil (MMF) or azathioprine at the time of TB diagnosis, in the preceding 3 months or in the past. For corticosteroids, the mode of administration, current dose, and cumulative dose at the time of TB diagnosis were recorded. The outcome was recorded as continuing follow-up, lost to follow-up, or died. We calculated the interval from the time of diagnosis of SLE to TB diagnosis in patients who developed TB. The control group comprised a similar number of patients with SLE but without TB, who were matched for disease duration, and their disease activity and treatment at the time of their last visit were recorded.

Ethical approval for the study was obtained from the Biomedical Research Ethics Committee of the University of KwaZulu-Natal (BE 223/16), the management of IALCH and the KwaZulu-Natal Department of Health.

### Statistical analysis

The data collected was analyzed using SPSS version 23 (IBM Corp, Armonk, NY, United States). Categorical variables are expressed as numbers (percentages). Depending on the normality test, numerical variables are represented as either means ± standard deviations (S.D.) or medians and interquartile range (IQR). The Pearson’s Chi-squared or Fisher’s exact test was used to test for association between categorical variables. The independent samples t-test or the Mann–Whitney *U*-test was used to test for equality of means or median values. Multiple logistic regression analysis was performed to identify independent risk factors for TB when comparing patients with SLE-TB and SLE controls, and for EPTB when comparing patients with EPTB vs. PTB. We also analyzed the cumulative SLE criteria to identify any independent risk factors for TB compared to controls, and for EPTB compared to PTB. The significance level was set at *p* ≤ 0.05 for all tests.

## Results

### Demographic data and prevalence of TB

There were 512 patients who fulfilled the SLICC criteria for SLE and did not have HIV infection or features of any other connective tissue diseases. The ethnicity of the patients was 282 (55.1%) Indian people, 184 (35.9%) Black African people, 28 (5.5%) admixed African people and 18 (3.5%) White people (19). The mean age at the diagnosis of SLE was 32.09 ± 14.13 years and 466 (91.0%) were females. There were 72 patients who were diagnosed with TB, representing a prevalence of 14.1% of our SLE patients. The prevalence of TB among the different ethnic groups was 12.8% (36/282) for Indian people, 15.8% (29/184) Black African people, 26.9% (7/26) admixed African people and none among the 18 White people. Among the patients with TB, 30/72 (41.7%) had PTB while 42/72 (58.3%) had EPTB.

### Comparison of the SLE patients with TB and SLE controls

#### Demographic data

The gender, ethnicity, age at diagnosis of SLE, duration of SLE, duration of follow up, the cumulative SLICC criteria, the SLEDAI 2 K score and outcome in our TB patients and controls are shown in Table 1. In the TB patients, the age at diagnosis of TB, interval from diagnosis of SLE to the TB, number of SLICC criteria at diagnosis of SLE and the SLEDAI 2-K score at diagnosis of TB are also shown in Table 1.

**Table 1:** Comparison of the demographic data, disease activity, treatment and outcome in SLE-TB patients and controls and patients with PTB and EPTB.

	Total TB *n* = 72 (%)	Controls *n* = 72 (%)	*p*-value	PTB *n* = 30 (%)	EPTB *n* = 42 (%)	*p*-value
Age at SLE diagnosis	31.3 ± 13.1	30.2 ± 13.22	0.574	35.2 ± 13.5	28.5 ± 12.3	**0**.**052**
(mean ± SD) years						
Age at TB diagnosis	36.3 ± 15.2			41.9 ± 15.4	32.3 ± 13.9	**0**.**010**
(mean ± SD) Years						
Interval from SLE to TB –	29.5			74	9.5	**<0**.**001**
median (IQR) months	(6.0–82.3)			(21.8–111.5)	(3.5–61.0)	
Duration of disease –	101	107	0.856	124	81.5	**0**.**003**
median (IQR) months	(53.3–157.8)	(56.5–160.0)		(77.8–190.5)	(20.5–116.8)	
Duration of follow-up –	77.5	91.5	0.072	83	58.5	**0**.**031**
median (IQR) months	(31.3–112.0)	(44.3–131.5)		(48.5–138.0)	(17.8–95.0)	
Gender (Female)	65 (90.3)	70 (97.2)	0.165	27 (90.0)	38 (90.5)	1.000
Black African people	29 (40.3)	30 (41.7)		10 (33.3)	19 (45.2)	
Indian people	36 (50.0)	39 (54.2)		17 (56.7)	19 (45.2)	
Admixed African people	7 (9.7)	1 (1.4)		3 (10.0)	4 (9.5)	
White people	0 (0.0)	0 (0.0)		0 (0.0)	0 (0.0)	
Total SLEDAI 2K -median (IQR)	8 (4.0–13.8)	0.0 (0.0–4.0)	**<0.001**	6 (3.8–9.3)	8 (5.5–16.0)	**0.022**
Common disease activity features						
Vasculitis	8 (11.1)	2 (2.8)	**0**.**049**	4 (13.3)	4 (9.5)	0.711
Arthritis	33 (45.8)	7 (9.7)	**<0**.**001**	16 (53.3)	17 (40.5)	0.280
Any renal	21 (29.2)	11 (15.3)	**0.045**	2 (6.7)	19 (45.2)	**<0.001**
Rash	18 (25.0)	6 (8.3)	**0**.**007**	7 (23.3)	11 (26.2)	0.783
Mucosal ulcers	14 (19.4)	5 (6.9)	**0**.**027**	4 (13.3)	10 (23.8)	0.268
Low complement	27 (37.5)	7 (9.7)	**<0**.**001**	8 (26.7)	19 (45.2)	0.109
Raised dS-DNA antibodies	22 (30.6)	2 (2.8)	**<0**.**001**	5 (16.7)	17 (40.5)	**0**.**031**
Thrombocytopenia	11 (15.3)	4 (5.6)	0.056	1 (3.3)	10 (23.8)	**0**.**021**
Immunosuppressive therapy						
Prednisone at TB/last visit (LV)	55 (76.4)	51 (70.8)	0.449	26 (86.7)	29 (69.0)	0.083
Cumulative prednisone dose in preceding 3 months (mg)	675	450	**0.037**	675	675	0.486
	(450–1.500)	(450–750)		(450–1425)	(450–1875)	
Intravenous MP^a^ ever	32 (44.4)	40 (55.6)	0.182	5 (16.7)	27 (64.3)	**<0**.**001**
Intravenous MP^a^ at TB/LV	3 (4.2)	0 (0.0)	1.000	0 (0.0)	4 (9.5)	0.135
Azathioprine ever	22 (30.6)	33 (30.6)	1.000	10 (33.3)	12 (28.6)	0.665
Azathioprine at TB/LV	7 (9.7)	6 (8.3)	0.771	3 (10.0)	4 (9.5)	1.000
MMF^b^ ever	26 (36.1)	23 (31.9)	0.598	2 (6.7)	24 (57.1)	**<0**.**001**
MMF at TB/LV	13 (18.1)	23 (31.9)	0.674	0 (0.0)	13 (31.0)	**<0**.**001**
Cyclophosphamide ever	18 (25.0)	12 (16.7)	0.218	4 (13.3)	14 (33.3)	0.053
Cyclophosphamide – at TB/LV	2 (2.8)	3 (4.2)	1.000	0 (0.0)	2 (4.8)	0.507
Immunosuppressive ever	47 (65.3)	36 (50.0)	0.064	13 (43.3)	34 (81.0)	**<0.001**
Immunosuppressive at TB/LV	20 (27.8)	21 (29.2)	1.000	3 (10.0)	17 (40.5)	**0.018**
Chloroquine ever	58 (80.6)	56 (77.8)	0.682	26 (86.7)	32 (76.2)	**0.268**
Chloroquine at TB/LV	43 (59.7)	46 (63.9)	0.394	18 (60.0)	23 (54.8)	**0.655**
Outcome						
Continuing follow up	53 (73.6)	44 (61.1)	0.110	23 (76.7)	30 (71.4)	0.183
Lost to follow up	11 (15.3)	20 (27.8)	0.068	5 (16.7)	6 (14.3)	1.000
Died	8 (11.1)	8 (11.1)	1.000	2 (6.7)	6 (14.3)	0.126

All the *p*-values are statistical significance tests which are shown in bold. ^a^MP, methylprednisolone; ^b^MMF, mycophenolate mofetil.

A comparison of the TB patients and controls showed that there was no difference in the mean age at the diagnosis of SLE (*p* = 0.574), median duration of disease in months (*p* = 0.856), gender (females 90.3% vs. 97.2%; *p* = 0.165), and proportion of Indian people (50.0% vs. 54.2%) and Black African people (40.3% vs. 41.7%).

#### Comparison of the clinical and laboratory features, treatment, and outcome between SLE-TB patients with SLE controls

The SLEDAI-2K scores and the components of the SLEDAI-2K which showed significant differences are shown in Table 1. Patients with TB had a significant increase in disease activity (SLEDAI-2K score) at the time of diagnosis of TB compared to controls at their last visit with a median score of 8 (IQR 4.0–13.75) vs. 0.0 (IQR 0.0–4.0; *p* < 0.001). The disease activity components which were significantly increased in patients with SLE-TB were arthritis (*p* < 0.001), skin rashes (*p* = 0.007), mouth ulcers (*p* = 0.027), renal involvement (*p* = 0.045), vasculitis (*p* = 0.049), and low complement (*p* < 0.001) and raised dS-DNA antibodies (*p* < 0.001).

The immunosuppressive treatment which the patients received at the time of diagnosis of TB, or at any stage of the disease is shown in Table 1. The median cumulative dose of prednisolone was higher in patients with TB than in control group (675 mg vs. 450 mg, *p* = 0.037). There was no significant difference in the use of immunosuppressive medication between the two groups as shown in Table 1.

A review of the outcome of the patients with SLE-TB and controls showed that there was no significant difference in the number of patients who were continuing follow up or had died, but an increased number of controls (27.8% vs. 15.3%; *p* = 0.068) were lost to follow up but this difference was not significant.

We undertook a multivariate logistic regression analysis using the variables which were significantly different between SLE -TB patients and controls. We found that the only independent predictor for TB among our SLE patients was the presence of arthritis (*p* = 0.030).

### Comparison of patients with PTB and EPTB

#### Demographic data

The results of the gender, ethnicity, age at diagnosis of SLE, age at diagnosis of TB, interval from diagnosis of SLE to the TB, duration of disease, duration of follow up, the SLEDAI 2-K score at diagnosis of TB and outcome for patients with PTB and EPTB are shown in Table 1.

Comparison of the 42 patients with EPTB and 30 patients with PTB showed that there was no difference in the proportion of females (*p* = 1.000). Patients with EPTB were younger at SLE diagnosis but the difference was not significant (*p* = 0.052). When compared to patients with PTB, the EPTB patients were significantly younger at diagnosis of TB (32.3 ± 13.9 vs. 41.9 ± 15.4 *p* = 0.010), and they had a shorter median interval between diagnosis of SLE and TB (9.5 vs. 74.0 months, *p* < 0.001). They also had a shorter median duration of disease (81.5 vs. 124.0, *p* = 0.003) and median duration of follow up (58.5 vs. 83.0, *p* = 0.031).

#### Comparison of the clinical and laboratory features, treatment, and outcome between patients with EPTB and PTB

Patients with EPTB had significantly higher disease activity scores compared to patients with PTB (*p* = 0.022) with SLEDAI scores of 8 (IQR 5.5–16.0) vs. 6 (IQR 3.8–9.3). The disease activity components which were significantly increased were renal disease (*p* < 0.001), thrombocytopenia (*p* = 0.021) and raised ds-DNA antibodies (*p* = 0.031). The EPTB patients also received more intravenous methylprednisolone (IV-MP) (64.3% vs. 16.7%; *p* < 0.001), MMF at TB diagnosis (31% vs. 0; p < 0.001) or ever received MMF (57.1% vs. 6.7%; p < 0.001).

The laboratory findings in patients with PTB and EPTB are shown in Table 2. The only significant abnormality between the groups was a higher median globulin level 46.0 (IQR 37.25–49.75) vs. 36.0 (IQR 31.5–42.0) g/l in patients with PTB (*p* < 0.001). Comparison of the outcome in Table 1 showed that even though there were more deaths among our EPTB patients, this difference was not significant (14.3% vs. 6.7%; *p* = 0.126).

**Table 2:** Comparison of the laboratory findings in patients with PTB and EPTB.

Laboratory results Median – (IQR)	PTB (*n* = 30)	EPTB (*n* = 42)	*p*-value	Total TB (*n* = 72)
Hemoglobin g/dL	9.85 (9.40–11.13)	10.20 (8.65–11.30)	0.902	9.95 (9.03–11.30)
White blood cells 10^9^ /L	6.96 (4.55–9.70)	6.64 (4.78–8.45)	0.635	6.69 (4.78–9.59)
Lymphocyte count 10^9^/L	0.92 (0.50–1.47)	0.67 (0.30–1.16)	0.498	0.69 (0.36–1.20)
Urea mmol/L	4.85 (2.83–7.80)	5.50 (4.48–9.43)	0.133	5.25 (3.60–8.85)
Creatinine umol/L	62.00 (51.00–86.00)	65.50 (52.50–123.00)	0.406	65.00 (52.00–112.00)
Erythrocyte sedimentation mm/h	95.50 (66.75–116.25)	60.00 (51.50–105.50)	0.092	71.00 (54.00–111.00)
C- reactive protein mg/L	71.30 (7.75–140.40)	60.00 (22.20–105.00)	0.943	64.00 (14.25–113.00)
Albumin g/L	32.00 (28.50–38.50)	33.00 (28.50–39.00)	0.806	32.50 (28.50–39.00)
Globulin g/L	46.00 (37.25–49.75)	36.00 (31.50–42.00)	**<0.001**	40.00 (34.00–46.00)
AST IU/L	32.00 (22.50–39.00)	22.00 (18.00–35.75)	0.290	23.00 (18.00–35.50)
ALT IU/L	17.50 (12.25–25.50)	20.00 (12.00–48.50)	0.480	19.50 (12.00–29.25)
GGT IU/L	41.00 (33.25–70.00)	50.50 (27.00–157.25)	0.372	44.50 (33.00–110.50)
Alkaline phosphatase IU/L	86.00 (69.50–96.75)	79.00 (61.00–175.00)	0.992	80.00 (66.00–115.00)
Urine Protein g/24 h	0.21 (0.13–2.00)	0.57 (0.29–2.79)	0.224	0.38 (0.21–2.32)
C3 gm/L	0.81 (0.36–0.91)	0.70 (0.48–1.30)	0.339	0.81 (0.45–1.10)
C4 gm/L	0.20 (0.08–0.23)	0.13 (0.07–0.28)	0.986	0.14 (0.07–0.24)

All the *p*-values are statistical significance tests which are shown in bold.

A multivariate logistic regression analysis using the significantly different variables on univariate analysis showed that the only independent predictors for EPTB were the ever use of MMF (*p* = 0.003) and ever use of IV-MP (*p* = 0.027).

### Comparison of the cumulative ACR criteria in SLE-TB patients vs. SLE controls and EPTB vs. PTB

A comparison of the cumulative SLICC criteria among patients with SLE-TB and SLE controls in Table 3 showed that acute cutaneous lesions were more common in controls (88.9% vs. 77.8%; *p* = 0.074) while antiphospholipid antibodies were more common in patients with TB (25.0% vs. 12.5%; *p* = 0.055) but these differences were not statistically significant.

**Table 3:** Comparison of the cumulative criteria in patients with SLE-TB and controls and patients with PTB and EPTB.

	Total TB*n* = 72 (%)	Control*n* = 72 (%)	*P*-value	PTB*n* = 30 (%)	EPTB*n* = 42 (%)	*p*-value
Clinical criteria	
1. Acute cutaneous lupus	56 (77.8)	64 (88.9)	0.074	26 (86.7)	30 (70.1)	0.125
2. Chronic cutaneous lupus	18 (25.0)	19 (26.4)	0.849	4 (13.3)	14 (33.3)	0.053
3. Oral ulcers	40 (55.6)	43 (59.7)	0.613	18 (60.0)	22 (52.4)	0.521
4. Non-scarring alopecia	24 (33.3)	20 (27.8)	0.469	9 (30.0)	15 (35.7)	0.612
5. Synovitis involving two or more joints	62 (86.1)	57 (79.2)	0.271	26 (86.7)	36 (85.7)	1.000
6. Serositis	24 (33.3)	17 (23.6)	0.196	8 (26.7)	16 (38.1)	0.310
7. Renal	38 (52.8)	31 (43.1)	0.243	8 (26.7)	30 (71.4)	**<0**.**001**
8. Neurologic	23 (31.9)	22 (30.6)	0.857	5 (16.7)	18 (42.9)	**0**.**019**
9. Haemolytic anemia	17 (23.6)	17 (23.6)	1.000	3 (10.0)	14 (33.3)	**0**.**022**
10. Leucopenia/lymphopenia	47 (65.3)	45 (62.5)	0.729	23 (76.7)	24 (57.1)	0.086
11. Thrombocytopenia	28 (38.9)	25 (34.7)	0.604	6 (20.0)	22 (52.4)	**0**.**005**
Immunological criteria		
1. ANA level high	71 (98.6)	72 (100)	1.000	29 (96.7)	42 (100)	0.128
2. Anti-dsDNA	48 (66.7)	41 (56.9)	0.230	17 (56.7)	31 (73.8)	0.417
3. Anti-Sm	17 (23.6)	19 (26.4)	0.700	7 (23.3)	10 (23.8)	0.963
4. Antiphospholipid antibody	18 (25.0)	9 (12.5)	0.055	5 (16.7)	13 (31.0)	0.168
5. Low complement	43 (59.7)	40 (55.6)	0.613	14 (46.7)	29 (69.0)	0.056
6. Direct Coomb’s test	13 (18.1)	13 (18.1)	1.000	0 (0.0)	13 (31.0)	**<0**.**001**

All the *p*-values are statistical significance tests which are shown in bold.

The EPTB patients had a significant increase in renal involvement (*p* < 0.001), neurologic manifestations (*p* = 0.019), hemolytic anemia (*p* = 0.022), positive Coomb’s test (*p* < 0.001) and thrombocytopenia (*p* = 0.005) compared to patients with PTB.

The multivariate logistic regression analysis showed that among the SLICC cumulative criteria, the presence of renal disease (*p* = 0.007) was the only independent risk factor for EPTB.

### Mode of diagnosis of tuberculosis and sites of extrapulmonary tuberculosis

The mode of diagnosis in patients with PTB was by sputum examination (microscopy, culture, and or GeneXpert) in 27 (90%) of 30 patients (Supplementary Table S1).

The diagnosis of EPTB was made by analysis of the specimens by microscopy, GeneXpert, culture and or histology in 25 patients. In the remaining 17 patients, diagnosis was based on history, clinical examination, laboratory tests, imaging studies and/or response to treatment.

The most common extra-pulmonary sites were pleural (10 patients), lymph nodes (8), pericardial (5), meningitis (3), and skin (3). Four patients had abscesses, two had joint involvement, two had urogenital involvement and one each had peritoneal, brain, liver, and bone marrow involvement.

## Discussion

Most of our patients were Black African people and Indian people and there was no significant difference in the prevalence of TB between them (Table 1). There are no epidemiological data on the prevalence of SLE in South Africa. The reasons for the higher proportion of Indian people in our study are multifactorial and include better access to care as most of them live in urban areas, socioeconomic factors, education, and cultural factors.

Tuberculosis is a common communicable disease and remains one of the leading causes of death worldwide. In 2014 and 2015, the WHO’s End TB Strategy was adopted by member countries of the WHO and United Nations (16). Most of the 30 high burden countries have 150–400 cases per 100,000 population, while South Africa is one of the few countries with an even higher burden of more than 500 cases per 100,000 population (17). In view of the high background prevalence of TB in our environment, we undertook this medical records review study to determine the prevalence of TB, proportion of patients with EPTB, identify risk factors for PTB and EPTB and compare our findings with other centers around the world.

There is a complex interplay between SLE and TB (21, 22). Patients with SLE are at increased risk of developing infections, including TB. In SLE patients, TB is more likely to be extrapulmonary, the pulmonary involvement, is more severe, and relapses occur more frequently (22).

Infections may also trigger autoimmune diseases and contribute to the induction and flares in SLE (21, 23). In Taiwan the prevalence of TB is higher in SLE, and TB is a risk factor for precipitating SLE (24). There was a history of TB in 20% of 70 SLE patients in India (25). A concurrent diagnosis of SLE and TB was made in 12 (16%) of the 76 patients in Hong Kong, 11 (22.9%) of 48 patients in North India, and 32 (12.9%) of 249 patients in China (26–28).

Establishing a diagnosis of TB in patients with SLE is often a challenging task as they share many similar manifestations including fever, weight loss and constitutional disturbances. In addition, manifestations such as serositis, pulmonary and neurological may occur in both SLE and TB. The diagnosis of latent TB is associated with additional challenges in SLE. The tuberculin skin test (TST) produces false negative results on immunosuppressive therapy and are of no value in countries with BCG (*Bacillus* Calmette-Guerin) vaccination programs. The interferon gamma release assays have improved the detection of latent TB but are less effective in identifying patients at risk of developing active disease (29).

In Table 4, we compare our findings with published reports of some of the larger studies from the different WHO regions around the world (6–15, 26–28, 30–41). We include the number of patients studied, number and percentage of patients with TB (including their age, gender, and interval from diagnosis of SLE to TB, where available), the number of SLE controls, and number and proportion of patients with EPTB. SLE was most common between age 25 and 35 years and majority of the patients were females.

**Table 4:** Comparison of the demographic data and prevalence of TB (PTB and EPTB) in SLE in different WHO regions.

First author surname	Country year	SLE patients number	TB prevalence *n* (%)	EPTB *n* (%)	Females *n* (%)	Age at SLE diagnosis Mean (SD) Median (IQR)	SLE to TB diagnosis Mean (SD) – (months)
Africa	
Current	South Africa	512	72 (14.1)	42 (58.3)	65 (90.3)	31.3 (13.1)	58.2 (73.8)
Hodkinson^a^ (9)	South Africa, 2009	568	97 (17.1)	28 (28.9)	86 (88.7)	33.7 (13.2)	
Region of Americas	
Chu (30)	United States, 2009	187	6 (3.2)	4 (66.7)	6 (100)		7.3 years
Pasoto (31)	Brazil, 2010	1,283	20 (1.6)	4 (20.0)	20 (100)	22.9 (6.3)	19.0 (9.4)
Torres-Gonzalez (8)	Mexico, 2018	5,365	72 (1.3)	48 (66.7)	60 (83.3)	24 (19–37)	6 (1–11) years
Gonzalez-Naranjo (10)	Colombia, 2021	268	67 (25.0)	36 (53.7)	56 (83.6)	28 (19–34)	
Southeast Asia	
Balakrishan (32)	India, 1998	146	17 (11.6%)	10 (58.8)			
Shyam (33)	India, 1996	309	41 (13.3)	9 (22.0)			
Muhammed (27)	India, 2021	1,335	48 (3.5)	37 (77.1)	39 (81.3)	27.6 (9.4)	3.0 (4.1) years
Ahmmed (34)	Bangladesh, 2019	230	23 (10.0)	8 (34.8)	16 (70.0)	27.6 (9.3)	4.3 (5.4) years
Hamijoyo (35)	Indonesia, 2017	813	93 (11.4)			27.7 (9.4)	
European region	
Sayarlioglu (13)	Turkey, 2004	556	20 (3.6)	9 (45.0)	17 (85)	32.2 (10)	46 (48)
Leon (36)	Spain, 2010	789	13 (1.6)	8 (61.5)	10 (77)	36 (11.2)	
Western Pacific	
Feng (15)	Singapore, 1982	311	16 (5.1)	9 (56)	15 (94)		
Yang (37)	Singapore, 2017	841	17 (5.0)	3 (17.6)			
Victorio- Navarra^b^ (12)	Philippines, 1996	390	54 (13.8)	38 (66.7)	53 (93)	32 (10)	
Kim (14)	South Korea, 1999	256	22 (8.8)	12 (54.5)	16 (72.7)	34 (24–67)	34 (3–180)
Yun (38)	South Korea, 2002	283	15 (5.3)	9 (60.0)	13 (86.7)	32.9 (11.7)	
Zhang (6)	China, 2008	452	42 (9.3)	31 (73,8)			
Zhang (6)	China, 2007	2,682	93 (3.5)	48 (51.6)			
Xiao (28)	China, 2021	10,469	249 (2.4)^e^	143 (57.4%)	202 (81.1)	33 (24,5–43)	9.6 (1.2–57.6)
Wang (39)	China, 2009	1,245	41 (3.3)		35 (85.4)	36.9 (14.0)	
Lao (40)	China, 2019	1,108	59 (5.3)	18 (30.5)	43 (73)	34.4 (12.7)	43.3 (45.6)
Liu (41)	China, 2021	2,959	41 (1.4)	20 (48.8)			
Tam (11)	Hong Kong, 2002	526	57 (10.8)	38 (66.7)	51 (89)		4.6 years
Mok^c^ (26)	Hong Kong, 2005	652	91 (14.0)	36 (39.6)			
Hou^d^ (7)	Taiwan, 2008	3,179	19 (0.66)	11 (52.4)	16 (84.2)	39.9 (16.7)	60.8 (60.8)

^a^111 TB episodes (81 PTB and 30 EPTB) in 97 patients; ^b^57 TB episodes (19 PTB 38EPTB) in 54 patients; ^c^91 episodes of TB in 76 patients; ^d^21 TB episodes (10 PTB and 11 EPTB) in 19 patients; ^e^this study included 33 patients with inactive TB.

There is a wide variation in the prevalence of TB in SLE around the world ranging from 0.66% in Taiwan to 25% in Colombia (7, 10). Some of the larger studies which report a low prevalence of TB are from Taiwan (0.66% of 3,179), Mexico (1.3% of 5,365), China (1.4% of 2,959 and 2.4% of 10,469) and Brazil (1.6% of 1,283) as shown in Table 4 (7, 8, 28, 31, 41). A higher prevalence was reported in Indonesia (11.4% of 813), Philippines (13.8% of 390), India (13.3% of 309), South Africa (17.1% of 568) and 14.1% of 512 in the current study (9, 12, 33, 35).

Table 4 shows that the prevalence of EPTB also varies with17.6% of 17 TB patients in Singapore (2017), 20% of 20 patients in Brazil (2010), 22% of 41 patients in India (1996), and 28.9% of 97 patients in Johannesburg (2009), South Africa (9, 31, 33, 37). We found a prevalence of EPTB of 58.2% among our 72 patients with TB and noted a high prevalence in some of the more recent series with 48.8% of 41 patients and 57.4% of 249 patients in two Chinese series (2021), 67% of 72 patients in Mexico (2018) and 54% of 67 patients in Colombia (2021) (8, 10, 28, 41). The prevalence of EPTB was between 50 and 60% in most of the studies in Table 4.

In Table 5 we compare the risk factors for SLE-TB patients compared to SLE controls. and the risk factors for EPTB vs. PTB (7–13, 15, 27, 28, 31, 34, 36–41, 42, 43). There are many variables which are identified on univariate analysis as risk factors for TB in SLE patients compared to SLE controls. The main factors are overall disease activity scores, disease activity of different organs and the cumulative dose and duration of corticosteroid therapy. We found high SLEDAI score as a risk factor for TB, similar to observations in Singapore, Philippines, and Bangladesh (12, 15, 34).The most common clinical feature was nephritis followed by arthritis, serositis, neurological and vasculitis in Table 5 (8, 11, 13, 15, 42). The laboratory findings associated with TB were lymphopenia, hypocomplementemia and raised ds-DNA antibodies. A common risk factor was the use of prednisone – in high cumulative dose, or for prolonged periods, and the use of IV-MP (Table 5).

**Table 5:** Comparison of the risk factors for TB vs. controls and for EPTB vs. PTB.

Surname of First Author, country, year, reference	SLE patients number	TB prevalence *n* (%)	EPTB*n* (%)	SLEcontrols	Risk factors for developing TB- SLE -TB *vs.* SLE controls – Univariate analysis.*- EPTB vs PTB in italics – Univariate analysis*- Multivariate regression analysis – independent risk factors in bold
Current, South Africa, 2022	512	72 (14)	42 (58)	72	SLE activity,**arthritis,** skin rashes, mouth ulcers, renal, vasculitis, low complement, raised dS-DNA antibodies, cumulative prednisone dose
					*EPTB: Renal, thrombocytopenia, raised ds-DNA antibodies, MMF at TB diagnosis, **ever MMF**, **ever use of intravenous methylprednisolone**.*
Hodkinson, South Africa, 2009 (9)	568	97 (17.1)	28 (29)	194	Black race, l**ymphopenia**, hypocomplementemia, neurological, intravenous corticosteroids (CS), **oral prednisone – maximum dose and duration**, use of immunosuppressive (IS) drugs.
Pasoto, Brazil 2010 (31)	1,283	20 (1.6)	4 (20)	40	Pleuritis
Torres-Gonzalez, Mexico, 2018 (8)	5,365	72 (1.3)	48 (67)	72	Renal,**Anemia, lymphopenia**, Hypocomplementemia, CYC pulse therapy, prednisone-current dose and 1-year**cumulative dose > 3 g**
					*EPTB: Malar rash, pleurisy/pericarditis, APLS, lymphopenia*
Gonzalez-Naranjo, Colombia, 2021 (10)	268	67 (25.0)	36 (54)	201	**Lymphopenia,** renal transplantation,**cumulative prednisone dose in preceding 12 months; ≥2 IS drugs in past 12 months**
Muhammed, India 2021 (27)	1,335	48 (3.5)	21 (43.8)	1,287	Male gender
					*EPTB: had lower absolute lymphocyte count at baseline*
Ahmmed, Bangladesh, 2019 (34)	230	23 (10.0)	8 (34.8)		High SLEDAI score (>12), prednisone intake >1,000 mg
Damara, Indonesia, 2022 (42)		24	8 (33.3)	24	Nephritis, cumulative prednisone dose, pulse CS, high SLEDAI score
Sayarlioglu, Turkey, 2004 (13)	556	20 (3.6)	9 (45)	96	Arthritis, renal involvement, cumulative dose and mean daily dose of prednisone before TB diagnosis
Leon, Spain, 2010 (36)	789	13 (1.6)	8 (62)	776	No significant differences for treatment – but high dose CS associated with more severe TB (Mortality in 30.8%).
Feng, Singapore, 1982 (15)	311	16 (5.1)	9 (56)		High disease activity and nephritis
Yang, Singapore, 2017 (37)	841	17 (5.0)	3 (17.6)		SLE independent predictor of TB
Victorio-Navarra^b^, Philippines, 1996 (12)	390	54 (13.8)	38 (67)	–	High SLEDAI and severity of disease index (SDI) with more extensive disease
Yun, S Korea, 2002 (38)	283	15 (5.3)	9 (60.0)	268	History of TB, longer duration of SLE and higher prednisone doses
Xiao, China, 2021 (28)	10,469	249 (2.4)	143 (57.4)	249	Arthritis, alopecia, mucocutaneous, musculoskeletal, higher dose of daily oral prednisone, intravenous pulse CS, cyclophosphamide
Lao, China, 2019 (40)	1,108	59 (5.3)	18 (31)		**Lymphopenia, anemia and cumulative dose of prednisone**
Liu, China, 2021 (41)	2,959	41 (1.4)	20 (48.8)	–	Previous History of TB, moderate / high doses of prednisone
Wang, China 2009 (39)	1,245	41 (3.3)		1,204	Increased serositis. (Renal disease was reduced patients with TB).
Zhang, China 2013 (43)		66	24 (36.4)	60	(TB associated with reduced disease activity and altered immune function - lower prevalence of antibodies, higher complement level and less severe hematological changes)
Tam, Hong Kong, 2002 (11)	526	57 (10.8)	38 (67)	114	Vasculitis, **Nephritis**, Organic brain syndrome, IV-MP, **Cumulative prednisone dose**
					*EPTB**–** More fever and shorter duration of symptoms*
Hou, Taiwan, 2008 (7)	3,179	19 (0.66)	11 (52)	–	*EPTB vs. PTB – No differences*

On multivariate analysis, the most common risk factors in the studies shown in Table 5 were the cumulative dose, and duration of corticosteroids, and lymphopenia, anemia, and nephritis.

There are fewer studies on the risk factors in patients with EPTB compared to PTB. In Table 5 we show the risk factors for EPTB identified in Mexico were malar rash, pleurisy/pericarditis and anti-phospholipid antibody syndrome and lymphopenia, in India absolute lymphopenia, in Hong Kong more fever and a shorter duration of symptoms, while no differences were noted in Taiwan (7, 8, 11, 27). We found that on univariate analysis, the risk factors for EPTB were higher disease activity scores, renal disease, thrombocytopenia, raised ds-DNA antibodies, ever use of IV-MP, and ever use of MMF, and MMF at TB diagnosis as shown in Table 1. However, on multivariate regression analysis, the only independent risk factors for EPTB were the ever use of MMF (*p* = 0.003) and IV-MP (*p* = 0.027). We note that while high disease activity and use of corticosteroids increase the risk of TB, it is possible that the extent of the disease activity (significantly higher for EPTB than PTB), and intensity of immunosuppressive therapy (greater IV-MP use for EPTB than PTB) which may contribute to the increased risk of EPTB.

In the earlier SLE studies, TB was diagnosed within 12 months of SLE diagnosis in 43% in Singapore, and 53% in India (15, 32).Our median interval between the diagnosis of SLE and development of TB was 29.5 (IQR 6.0–82.3) months but there was a significantly lower interval in patients with EPTB of 9.5 (IQR 3.5–61.0) compared to 74.0 (IQR 21.8–111.5) (*p* = 0.010) for PTB. Table 5 shows that the mean or median interval between diagnosis of SLE and the development of TB was between 3 and 5 years for most of the studies except for a median duration of 9.6 (1.2–57.6) months by Xiao in China and a mean of 19.4 ± 9.4 months in Brazil (28, 31). A possible explanation for the early onset of TB after SLE diagnosis could be a delay in referral or diagnosis, resulting in presentation with severe acute multiorgan involvement which requires intensive immunosuppression and increases the risk of infections, including TB.

Even with progress towards the WHO and UN End TB strategy, many exposed people in endemic areas will be at risk of reactivation of TB with autoimmune diseases or immunosuppressive medication (44). A multidisciplinary approach is required to identify patients at increased risk of TB who will benefit from prophylactic therapy.

In conclusion, we report a 14.1% prevalence of TB (with EPTB in 58.3%) in a multi-ethnic cohort of patients with SLE seen in a single center in a TB endemic area in Durban, South Africa. The risk factors in SLE patients who developed TB compared to SLE controls were an increased prevalence of clinical and laboratory measures of disease activity and increase in the cumulative prednisone use over the preceding 3 months. Compared to PTB patients, the EPTB also had an increase in features of disease activity and ever use of IV-MP and MMF, with the ever use of MMF and IV-MP being the only risk factors on multivariate analysis. Renal involvement was the only cumulative criterion that was a risk factor for EPTB. Despite the limitations of a medical records review study with the lack of a standardized protocol and missing data, we believe this study is timely to raise further evidence of the burden of TB in SLE while the global community works towards the WHO and United Nations End TB strategy.

## Data availability statement

The original contributions presented in the study are included in the article/Supplementary materials, further inquiries can be directed to the corresponding author.

## Ethics statement

The studies involving human participants were reviewed and approved by Biomedical Research Ethics Committee of the University of KwaZulu-Natal – number BE 223/16. Written informed consent from the participants’ legal guardian/next of kin was not required to participate in this study in accordance with the national legislation and the institutional requirements.

## Author contributions

KA-a: design of the study, acquisition, analysis and interpretation of the data, preparation of the draft, preparation of the final submission, accountable for all aspects of the study. NM: design of the study, interpretation of the data, revision of the draft, review of the final submission, accountable for all aspects of the study. GM: conception and design of the study, analysis and interpretation of the data, revision of the draft, review of the final submission, and accountable for all aspects of study. All authors contributed to the article and approved the submitted version.

## Conflict of interest

The authors declare that the research was conducted in the absence of any commercial or financial relationships that could be construed as a potential conflict of interest.

## Publisher’s note

All claims expressed in this article are solely those of the authors and do not necessarily represent those of their affiliated organizations, or those of the publisher, the editors and the reviewers. Any product that may be evaluated in this article, or claim that may be made by its manufacturer, is not guaranteed or endorsed by the publisher.
