# Supplementary material for: Tuberculosis remains a major burden in systemic lupus erythematosus patients in Durban, South Africa
Source: Front Med (Lausanne). 2023 Mar 1;10:1118390. doi: 10.3389/fmed.2023.1118390 (PMC10014752; doi:10.3389/fmed.2023.1118390)
Supplement: Supplementary file 1 [file Table_1.pdf]

Supplementary Table S1. Mode of diagnosis in patients with pulmonary and extra-pulmonary tuberculosis

MODE OF DIAGNOSIS	**PTB n=30**			**EPTB n=42**		
	Number	Alone / Combination		Number	Alone / Combination	
Microscopy	14	2 / 12	+ 5G, 2GC, 5C	10	1 / 9	1G, 1GCH, 1GH, 2C,1CH,3H
GeneXpert	16	5 / 11	+ 5M, 2MC, 4C	4	0 / 4	1MCH, 1MH, 1M, 1C
Culture	15	4 / 11	+ 5M, 2MG, 4G	14	6 / 8	1MGH, 2M, 1MH, 1G, 3H
Histology	-	-	-	14	5 / 9	1MGC, 3M, 1MC, 1MG, 3C
Clinical diagnosis	3	3		17	17	

G - GeneXpert; C - culture; M - microscopy; H – histology
